# Reliability and validity of the Chinese version of the pediatric quality of life inventory^TM^ (PedsQL^TM^) 3.0 neuromuscular module in children with Duchenne muscular dystrophy

**DOI:** 10.1186/1477-7525-11-47

**Published:** 2013-03-15

**Authors:** Jun Hu, Li Jiang, Siqi Hong, Li Cheng, Min Kong, Yuanzhen Ye

**Affiliations:** 1 Ministry of Education Key Laboratory of Child Development and Disorders, Children’s Hospital, Chongqing Medical University, No. 136 Zhongshan Er Road, Yuzhong District, Chongqing, China; 2 Key Laboratory of Pediatrics in Chongqing, Children’s Hospital, Chongqing Medical University, No. 136 Zhongshan Er Road, Yuzhong District, Chongqing, China; 3 Chongqing International Science, Technology Cooperations Center for Child Development and Disorders, Children’s Hospital, Chongqing Medical University, No. 136 Zhongshan Er Road, Yuzhong District, Chongqing, China; 4 Department of Neurology, Children’s Hospital, Chongqing Medical University, No. 136 Zhongshan Er Road, Yuzhong District, Chongqing, China

**Keywords:** Duchenne muscular dystrophy, Pediatric quality of life inventory^TM^, Neuromuscular module, Chinese version, Reliability, Validity

## Abstract

**Background:**

The Pediatric Quality of Life Inventory^TM^ (PedsQL^TM^) is a widely used instrument to measure pediatric health-related quality of life (HRQOL) in children aged 2 to 18 years. The current study aimed to evaluate the reliability and validity of the Chinese version of the PedsQL^TM^ 3.0 Neuromuscular Module in children with Duchenne muscular dystrophy (DMD).

**Methods:**

The PedsQL^TM^ 3.0 Neuromuscular Module was translated into Chinese following PedsQL^TM^ Measurement Model Translation Methodology. The Chinese version scale was administered to 56 children with DMD and their parents, and the psychometric properties were evaluated.

**Results:**

The missing value percentages for each item of the Chinese version scale ranged from 0.00% to 0.54%. Internal consistency reliability approached or exceeded the minimum reliability standard of α = 0.7 (child α = 0.81, parent α = 0.86). Test-retest reliability was satisfactory, with intraclass correlation coefficients (ICCs) of 0.66 for children and 0.88 for parents (*P* < 0.01). Correlation coefficients between iteims and their hypothesized subscales were higher than those with other subscales (*P* < 0.05). The subscale of “About My/My Child’s Neuromuscular Disease” significantly related to mobility and stair climbing status (Child *t* = 2.21, Parent *t* = 2.83, *P* < 0.05). The inter-correlations among the Chinese version of the PedsQL^TM^ 3.0 Neuromuscular Module and the PedsQL^TM^ 4.0 Generic Core Scales had medium to large effect sizes (*P* < 0.05). The child self-report scores were in moderate agreement with the parent proxy-report scores (ICC = 0.51, *P* < 0.05).

**Conclusions:**

The Chinese version of the PedsQL^TM^ 3.0 Neuromuscular Module has acceptable psychometric properties. It is a reliable measure of disease-specific HRQOL in Chinese children with DMD.

## Background

Duchenne muscular dystrophy (DMD) is the most common form of muscular dystrophy in childhood and affects 1:3500 live male births [1]. DMD is caused by mutations (typically deletions) in the dystrophin gene on the X chromosome [2]. Since the gene was cloned in 1986, many strategies for treatment have been proposed and tested in animal models, as well as human subjects [3]. Although there are no specific DMD treatments, corticosteroid, nutrition, respiratory, cardiac, orthopedic, rehabilitative, and psychosocial interventions have improved function, quality of life, health, and longevity. Thus, children diagnosed with DMD may be able to reach the fourth decade of life, which is a significant improvement over the historical DMD life expectancy [4-8].

Health-related quality of life (HRQOL) is increasingly acknowledged as an important health outcome measure in clinical trials and health services research and evaluation involving children with neuromuscular disorders [9-12]. However, one challenge in measuring pediatric HRQOL is that the instrument must account for physical, emotional, social, and cognitive development that takes place during childhood and adolescence. In addition, questionnaires should provide the required information [13]. There is an emerging perspective that both generic and disease-specific HRQOL measures should be administered to pediatric patients with chronic health conditions in order to comprehensively evaluate patient HRQOL [14,15].

The Pediatric Quality of Life Inventory (PedsQL^TM^) Measurement Model was designed to integrate the relative merits of a generic core instrument with disease-specific modules [11,12,16-19]. The PedsQL^TM^ Measurement Model was designed to develop and test brief measures for the broadest age group empirically feasible, including child self-report for young children [20,21]. The PedsQL^TM^ 4.0 Generic Core Scales was specifically intended for application in healthy and patient populations [22]. It has been translated into many languages and widely used in more than 60 countries [23]. The Chinese version of the PedsQL^TM^ 4.0 Generic Core Scales has been developed and psychometrically evaluated [24], but the Chinese version PedsQL^TM^ 3.0 Neuromuscular Module for measuring HRQOL dimensions in children ages 2 to 18 years with neuromuscular disorders [10-12] has not been assessed for reliability and validity. The aim of the current study was to investigate the psychometric properties of the Chinese version of the PedsQL^TM^ 3.0 Neuromuscular Module in children with DMD for use in conjunction with the Chinese version of the PedsQL^TM^ 4.0 Generic Core Scales.

## Methods

### Subjects

This study was carried out in China between December 2009 and December 2011. Children aged 2 to 18 years with DMD and their parents were recruited through the Children’s Hospital, Chongqing Medical University. The diagnosis of DMD was initially based on their clinical history and neuromuscular findings and was confirmed by DNA testing or muscular biopsy. Children were excluded from this study if they had other chronic diseases or major developmental disorders. Families who did not agree to participate were also excluded. A total of 56 families were included, and informed consent forms were signed by all subjects and their parents. It was approved in compliance with the Helsinki Declaration by the Ethics Committee of Children's Hospital, Chongqing Medical University. The average age of the 56 male DMD patients was 7.54 (standard deviation [SD] = 4.06; range 2–13 years. Six were ages 2–4, 18 were ages 5–7, and 32 were ages 8–13. The sample was classified into two grades using the Vignos scale for lower extremities [25]. Thirty-seven (66.07%) children could climb stairs, but 19 could not.

### Measures

#### PedsQL^TM^ 3.0 neuromuscular module

The module encompasses three scales: 1) About My/My Child’s Neuromuscular Disease (17 items), 2) Communication (3 items), and 3) About Our Family Resources (5 items) [11,12]. The scale is comprised of parallel child self-report and parent proxy-report formats for children aged 5 to 18 years, and a parent proxy-report format for children ages 2 to 4 years. It is divided into seven forms, including child self-report formats for ages 5–7, 8–12, and 13–18 and parent proxy-report formats for ages 2–4, 5–7, 8–12, and 13–18. The young child form (ages 5–7 years) does not contain the “Communication” or “About Our Family Resources” Scale. Items in all forms are essentially identical but contain slightly different language for first or third person tense. The participants are asked how much of a problem each item had been during the past month. Responses are rated on a 5-point Likert scale across child self-report for children, teens, and parent proxy-reports (0 = never a problem, 1 = almost never a problem, 2 = sometimes a problem, 3 = often a problem, 4 = almost always a problem). Items are linearly transformed to a 0 to 100 scale (0 = 100, 1 = 75, 2 = 50, 3 = 25, and 4 = 0) so that higher scores indicate better HRQOL. Scale scores are computed as the sum of the items divided by the number of items answered. If more than 50% of the items in the scale are missing, the scale score is not computed [26].

#### PedsQL^TM^ 4.0 generic core scales

The 23-item PedsQL^TM^ 4.0 Generic Core Scales encompass: 1) Physical Functioning (8 items), 2) Emotional Functioning (5 items), 3) Social Functioning (5 items), and 4) School Functioning (5 items) [20,22,24]. The formats, instructions, Likert scales, and scoring methods are the same as those of the PedsQL^TM^ 3.0 Neuromuscular Module. To create the Psychosocial Health Summary Score, the mean is computed as the sum of the items divided by the number of items answered in the Emotional, Social, and School Functioning Subscales.

### Procedure

The PedsQL^TM^ Measurement Model Translation Methodology was strictly followed in the linguistic translation process of the PedsQL^TM^ 3.0 Neuromuscular Module in this study [27]. It is summarized as the procedure of Forward Translation - Backward Translation – Preliminary Test - Field Test. The instruments were independently translated into Chinese by a pediatrician and a medical English teacher, both of whom were fluent in English, and a multidisciplinary team reviewed both drafts. A single reconciled Chinese version was developed after discussion. It was translated back into English by a bilingual pediatrician. Then, a comparison between the versions was made to detect any misunderstandings or mistranslations, and a second Chinese version was written [24]. Cognitive debriefing was conducted with six children with DMD and their parents to confirm that the final Chinese version was understandable and acceptable. Reports for all stages were sent to and accepted by Mapi Research Institute in Lyon, France, on behalf of Dr. James W. Varni, the copyright owner of the PedsQL^TM^.

### Data collection

Three physicians were trained as interviewers before the formal start of investigation. Participants completed the Chinese version of the PedsQL^TM^ 3.0 Neuromuscular Module and the PedsQL^TM^ 4.0 Generic Core Scales separately during hospitalization or an outpatient department visit. Paper-and-pencil questionnaires were self-administered for parents and for children ages 8 to 18 years and interview-administered for children ages 5–7 years. The interviewers were available to assist if the parents/children had questions on semantic or conceptual understanding. In the 56 parent questionnaires, 43 were completed by both parents together at the same time, and 13 by a single parent only. For test-retest reliability, a subset of (n =30) completed the PedsQL^TM^ measures a second time 4–6 weeks later during a routinely scheduled clinic visit if they had suffered no adverse events since the first visit.

### Data analysis

Data were analyzed with SPSS 17.0 for Windows. Descriptive analysis was used to report the demographic characteristics of children with DMD. The presence of floor and ceiling effects, which was the percentage of scores at the extremes and provided information on the distribution in a scale, was assessed for the Chinese version of the PedsQL^TM^ 3.0 Neuromuscular Module. Continuous variables are presented as mean and standard deviation (mean ± *SD*). Categorical variables are shown as observed frequencies and proportions. Feasibility was determined from the percentage of missing values [28]. Scale internal consistency reliability for the Chinese version scale was determined at baseline by calculating Cronbach’s alpha coefficient [29]. Scales with reliabilities ≥0.70 are recommended for comparing patient groups, while a reliability criterion of 0.90 is recommended for analyzing individual patient scores [30,31].

Test-retest reliability for the Chinese version scale was assessed for a subset of the sample (n = 30) using intraclass correlation coefficients (ICCs) [32,33]. Patients and their parents were assessed an average of 35.82 days (SD = 10.36) after baseline. ICCs ≤0.40 were designated as poor to fair agreement, 0.41-0.60 moderate agreement, 0.61-0.80 good agreement, and 0.81-1.00 excellent agreement [32-34].

Multitrait scaling analysis for the Chinese version scale was conducted at baseline using Pearson correlation analysis to determine the item-subscale correlations. Good scaling success was indicated if the correlation between an item and its hypothesized subscale was stronger than those with other subscales [35].

Construct validity for the Chinese version scale was determined utilizing the known-groups method [33], which compares scale scores across groups known to differ in the health construct being investigated. Because of the small number of patients in one category, we used an independent sample *t*-test to compare the ability to climb stairs (yes vs. no) with the baseline Neuromuscular Module scores to perform the construct validity. Based on previous findings regarding the association with patient HRQOL, we hypothesized that the “About My/My Child’s Neuromuscular Disease” subscale would be correlated to stair climbing status, with higher QOL higher for patients who could climb stairs compared with those who could not [11,12].

Additional construct validity was examined through an analysis of intercorrelations among Neuromuscular Module Scores, Generic Core Scales, and Summary Scores [30]. We hypothesized greater disease-specific symptoms or problems would correlate with lower overall generic HRQOL based on the conceptualization of disease-specific symptoms as causal indicators of generic HRQOL [36]. Pearson Product Moment Correlation coefficients are designated as small (0.10-0.29), medium (0.30-0.49), and large (≥0.50) [37].

Agreement between child self-reports and parent proxy-reports of the Chinese version scale was determined through ICCs [38], which offers an index of absolute agreement because it takes into account the ratio between subject variability and total variability [38,39].

## Results

### Descriptive analysis

A total of 50 children and 56 parents completed the Chinese version of the PedsQL^TM^ 3.0 Neuromuscular Module questionnaire, and the average time was about 5 minutes. Table 1 displays the means, standard deviations, and floor and ceiling effects for each subscale score. Ceiling effects in both child self- and parent proxy-reports ranged from 1.9-24.7%, with highest values in the “About My/My Child’s Neuromuscular Disease” subscale. Floor effects ranged from 1.2-14.6%, with a notably high value for the “Communication” subscale.

**Table 1:** **Reliability and descriptive statistics of the Chinese version of the PedsQL**^TM^** 3.0 neuromuscular module for DMD**

***Scale***	***n***	***α***	***Mean***	***SD***	***% floor***	***% ceiling***
*Child self-report*						
Total scale	32	0.81	53.64	10.55	2.7	19.5
About My Neuromuscular Disease	50	0.87	71.24	12.06	1.3	24.7
Communication	32	0.80	39.06	22.54	16.7	1.9
About Our Family Resources	32	0.73	52.50	15.76	1.7	3.8
*Parent proxy-report*						
Total scale	56	0.86	52.88	9.34	2.9	16.2
About My Child’s Neuromuscular Disease	56	0.87	71.06	11.23	1.2	22.5
Communication	56	0.83	37.50	20.41	14.6	2.1
About Our Family Resources	56	0.85	50.09	15.06	1.6	3.3

Note: Higher values denote better HRQOL.

% Floor/% Ceiling, the percentage of scores at the extremes of the scaling range; α, Cronbach’s alpha coefficient; SD, standard deviation.

### Feasibility

The percentage of missing item responses for all scales was 0.54% and 0.31% for the child self-report and parent proxy-reports, respectively.

### Internal consistency reliability

Internal consistency reliability coefficients are presented in Table 1. All child self-report and parent proxy-report scales exceeded the minimum reliability standard of 0.70 required for group comparisons.

### Test-retest reliability

ICCs for test-retest reliability of the Chinese version scale are presented in Table 2. The majority of these ICCs had good to excellent reliability.

**Table 2:** **ICCs for test-retest reliability of the Chinese version of the** PedsQL^TM^**3.0 neuromuscular module for DMD**

***Scale***	***Child self-report test-retest reliability ICC***	***Parent proxy-report test-retest reliability ICC***
Total scale	0.66^Δ^	0.88^Δ^
About My/My Child’s Neuromuscular Disease	0.59^Δ^	0.85^Δ^
Communication	0.48^*^	0.76^Δ^
About Our Family Resources	0.59^Δ^	0.80^Δ^

Note: ^*^P < 0.05, ^Δ^*P* < 0.01.

ICC, intraclass correlation designated as ≤ 0.40 poor to fair agreement; 0.41-0.60 moderate agreement; 0.61-0.80 good agreement; and 0.81-1.00 excellent agreement.

### Item-subscale correlations

Pearson correlation coefficients between items and subscale scores are presented in Table 3. We found that most items had moderate to strong correlations with their hypothesized subscales, which were higher than those with other subscales (*P* < 0.05).

**Table 3:** **Item-subscale correlations of the Chinese version of the PedsQL**^TM^**3.0 neuromuscular module for DMD**

***Subscales & Items***	***Child self-report***			***Parent proxy-report***		
	***AN***	***C***	***FR***	***AN***	***C***	***FR***
About My/My Child’s Neuromuscular Disease (AN)						
It is hard to breathe	0.05	0.02	0.05	0.12	0.17	0.04
Get sick easily	0.05	0.18	0.22	0.09	0.14	0.21
Get sores and/or rashes	0.29	0.17	0.03	0.27	0.19	0.10
Legs hurt	0.73	0.37	0.15	0.78	0.30	0.65
Feel tired	0.80	0.14	0.21	0.77	0.12	0.51
Back feel stiff	0.59	0.16	0.01	0.63	0.29	0.43
Wake up tired	0.74	0.04	0.00	0.71	0.16	0.56
Hands are weak	0.63	0.28	0.13	0.62	0.15	0.54
Hard to use the bathroom	0.71	0.43	0.31	0.69	0.36	0.30
Hard to gain or lose weight when one wants to	0.65	0.10	0.13	0.68	0.25	0.57
Hard to use my hands	0.69	0.31	0.03	0.78	0.07	0.56
Hard to swallow food	0.11	0.20	0.15	0.01	0.46	0.04
Takes a long time to bathe or shower	0.70	0.00	0.07	0.71	0.25	0.56
Get hurt accidentally	0.59	0.20	0.01	0.59	0.08	0.29
Take a long time to eat	0.48	0.14	0.19	0.48	0.21	0.28
Hard to turn oneself during the night	0.70	0.08	0.03	0.69	0.39	0.47
Hard to go places with one’s equipment	0.66	0.22	0.36	0.61	0.46	0.37
Communication (C)						
Hard to tell the doctors and nurses how I feel	0.28	0.81	0.19	0.26	0.83	0.18
Hard to ask the doctors and nurses questions	0.09	0.87	0.28	0.35	0.89	0.12
Hard to explain the illness to other people	0.13	0.87	0.16	0.21	0.87	0.02
About Our Family Resources (FR)						
Hard for my family to plan activities like vacations	0.13	0.18	0.53	0.55	0.17	0.69
Hard for my family to get enough rest	0.12	0.23	0.76	0.54	0.04	0.80
I think money is a problem in our family	0.03	0.19	0.82	0.46	0.10	0.87
I think my family has a lot of problems	0.01	0.17	0.84	0.64	0.06	0.79
Do not have the equipment one needs	0.05	0.14	0.70	0.52	0.09	0.80

Note: Pearson’s product moment correlations are designated as small (0.10), medium (0.30), and large (0.50).

Underlined values represent correlations between items and their hypothesized subscales.

AN, About My Neuromuscular Disease; C, Communication; FR, About Our Family Resources.

### Construct validity

Construct validity was assessed by comparing stair climbing mobility groups (climb stairs and non-climb) and is presented in Table 4. The a priori hypothesis that “About My/My Child’s Neuromuscular Disease” subscale would be significantly related to mobility was confirmed for both child self-report and parent proxy-report. The means increased for children who could climb stairs and those who could not for all of the Chinese version subscale scores. The inter-correlations between the Generic Core Scales and Summary Scores with the Neuromuscular Module are shown in Table 5. The majority of intercorrelations were in the medium to large range, supporting construct validity (*P* < 0.05).

**Table 4:** **The known-groups method test comparing stair climbing status of the Chinese version of the PedsQL**^TM^**3.0 neuromuscular module for DMD**

***Scale***	***Can climb stairs***		***Cannot climb stairs***		***Independent sample t-test***	
	***n***	***Mean (SD)***	***n***	***Mean (SD)***	***t***	***P***
Child self-report						
Total score	13	57.58 (11.24)	19	50.42 (8.20)	2.07	0.05
About My Neuromuscular Disease	31	75.49 (11.85)	19	68.15 (11.44)	2.21	0.03
Communication	13	43.33 (22.97)	19	35.29 (22.15)	1.01	0.32
About Our Family Resources	13	54.00 (12.98)	19	51.18 (18.16)	0.5	0.62
Parent proxy-report						
Total score	37	55.66 (7.89)	19	48.26 (12.10)	2.50	0.02
About My Child’s Neuromuscular Disease	37	74.16 (10.34)	19	65.90 (10.97)	2.83	0.01
Communication	37	40.24 (19.85)	19	32.94 (21.00)	1.30	0.20
About Our Family Resources	37	52.57 (11.72)	19	45.95 (19.01)	1.62	0.11

**Table 5:** **Pearson’s product moment correlations among the Chinese version of the PedsQL**^TM^**Scales for DMD**

***Scale***	***TG***	***PH***	***PsyH***	***EF***	***SF***	***SchF***	***TN***	***AN***	***C***	***FR***
**PedsQL**^**TM **^**Generic Core Scales**										
Total Generic Core (TG)	---	0.43	0.56	0.60	0.67	0.74	0.67	0.14	0.61	0.28
Physical Health (PH)	0.47	---	0.55	0.44	0.43	0.50	0.43	0.28	0.22	0.25
Psychosocial Health (PsyH)	0.61	0.49	---	0.50	0.72	0.61	0.28	0.14	0.55	0.45
Emotional Functioning (EF)	0.41	0.33	0.31	---	0.48	0.38	0.48	0.10	0.50	0.12
Social Functioning (SF)	0.78	0.56	0.40	0.65	---	0.26	0.40	0.15	0.68	0.51
School Functioning (SchF)	0.67	0.46	0.60	0.61	0.66	---	0.24	0.22	0.49	0.37
**PedsQL**^**TM **^**Neuromuscular Module**										
Total Neuromuscular Module (TN)	0.60	0.52	0.50	0.55	0.58	0.57	---	0.25	0.44	0.25
About My/My Child’s Neuromuscular Disease (AN)	0.39	0.38	0.23	0.46	0.30	0.40	0.30	---	0.20	0.27
Communication (C)	0.39	0.66	0.41	0.27	0.45	0.36	0.45	0.27	---	0.01
About Our Family Resources (FR)	0.11	0.01	0.21	0.04	0.18	0.34	0.18	0.10	0.20	---

Note: Pearson’s product moment correlations for child self-report and parent proxy-report are presented above and below the diagonal dashed lines, respectively.

Pearson’s product moment correlations are designated as small (0.10), medium (0.30), and large (0.50).

Underlined values represent correlations between child self-report and parent proxy-report subscales.

TG, Total Generic Core; PH, Physical Health; PsyH, Psychosocial Health; EF, Emotional Functioning; SF, Social Functioning; SchF, School Functioning; TN, Total Neuromuscular Module; AN, About My Neuromuscular Disease; C, Communication; FR, About Our Family Resources.

### Parent/child agreement

ICCs of the Chinese version scale are presented in Table 6. Most inter-correlations of subscales between child self-report and parent proxy-report were in the moderate agreement range. The greatest overall agreement was observed for the Total scale (0.51).

**Table 6:** **ICCs of the Chinese version of the PedsQL**^TM^**3.0 neuromuscular module for DMD**

***Scale***	***Parent–child agreement ICC***
Total scale	0.51^*^
About My/My Child’s Neuromuscular Disease	0.49^Δ^
Communication	0.21
About Our Family Resources	0.49^*^

Note: ^*^P < 0.05, ^Δ^*P* < 0.01.

ICC = intraclass correlation, ICCs are designated as ≤ 0.40 poor to fair agreement, 0.41-0.60 moderate agreement, 0.61-0.80 good agreement, and 0.81-1.00 excellent agreement.

## Discussion

The PedsQL^TM^ 3.0 Neuromuscular Module is a PedsQL^TM^ disease-specific modules designed to measure the neuromuscular specific HRQOL of pediatric patients. It has satisfactory psychometric properties for both the child self-report and the parent proxy-report and is appropriate for children in a broad age range. The data described here support the feasibility, reliability, and validity of the Chinese version of the PedsQL^TM^ 3.0 Neuromuscular Module in children with DMD living in mainland China. To our knowledge, it is also the first time that the PedsQL^TM^ 3.0 Neuromuscular Module was tested on Chinese children with DMD.

There were minimal missing item responses in the Chinese scale, indicating that children and their parents were able to provide good-quality data regarding pediatric HRQOL. However, our results show that there were several items, i.e., “hard to gain or lose weight when one wants to” and “do not have the equipment one needs,” with missing responses. Notably, the percentage of missing values was primarily attributable to 2–7 year old children. The reason may be that younger children had difficulty understand these questions, that most of them could not gain or lose weight, or that they did not require equipments because they were still able to walk. Regardless, this finding is consistent with those of the prior studies with other PedsQL^TM^ disease-specific modules [40] and indicates that some modifications for items of these subscales are necessary in toddler and young child versions. In addition, the highest ceiling effect was observed in the “About My/My Child’s Neuromuscular Disease” subscale. It is understandable in a mixed participant with different disease severity, where the majority of children with DMD (66.07%) could climb stairs which would be expected to influence markedly their daily lives. There was also a notable floor effect in the “Communication” subscale. It suggests that children with DMD lack good communication; this problem requires the joint efforts of the clinicians and the community.

Internal consistency reliabilities of the Chinese version exceeded the minimum alpha coefficient standard of 0.70 required for group comparisons for all child self-report and parent proxy-report scales. These findings are consistent with reliability estimates for the original English version [11,12], indicating acceptable reliability of the Chinese version scale. Although Cronbach’s alpha represents the lower limit of reliability of a measurement instrument and is a conservative estimate of actual reliability [41], scales that did not meet the 0.70 standard should only be used for descriptive analyses. Overall, responses to the Chinese version scale were in the good to excellent agreement range across a 4–6 week period and correlated significantly, supporting test-retest reliability and demonstrating that the Chinese version scale is stable over time.

In order to evaluate the item-subscale correlations of the Chinese version scale, we analyzed Pearson correlations between items and subscale scores. The results of correlations between items and their hypothesized subscales were high, but those between items and other subscales were weak, indicating good scaling success for child self-reports and parent proxy-reports.

Construct validity was assessed with the Known-Groups Method of comparing mobility groups (climb stairs vs. non-climb). The a priori hypothesis that “About My/My Child’s Neuromuscular Disease” subscale would be significantly related to mobility was confirmed for both child self-report and parent proxy-report. The subscales of “Communication” and “About Our Family Resources” showed unqualified discriminate abilities, probably due to the relatively small sample size, and both are recommended for further testing.

Construct validity was further tested by analyzing inter-correlations among the Chinese version scale Scores and the PedsQL^TM^ 4.0 Generic Core Scales and Summary Scores. Consistent with the conceptualization of disease-specific symptoms as causal indicators of generic HRQOL, the majority of inter-correlations among the Chinese version scale and the Generic Core Scales were in the medium to large range, supporting construct validity. Regarding the agreement between child self-report and parent proxy-report of the Chinese version scale, our data were mostly in the moderate agreement range. This is consistent with both the adult and pediatric literature, suggesting that information provided by proxy-respondents is not equivalent to that reported by patients [42,43]. Imperfect agreement between child self-report and parent proxy-report has been consistently documented in HRQOL measurements of children with and without chronic illness [44,45], particularly for less observable or internal symptoms.

This study has several limitations that should be considered when interpreting the results. In mainland China, many children with DMD have refused or abandoned treatment for financial reasons, and HRQOL information about these patients is lacking. All of the subjects in this study were recruited in a large city (Chongqing) in mainland China; determining whether the scale can be generalized to other regions will require further studies in other areas. Information on non-participants’ and participants’ socioeconomic status was not available and could affect our findings. The test-retest data were obtained with an interval of around 4–6 weeks, which was considered to be long enough to make the children with DMD and their parents forget their answers from the first test. However, a potential limitation of the long interval was the possibility of the occurrence of positive or negative events affecting the participants. In addition, the small sample size did not allow us to perform a factor analysis; correlation coefficients from factor analyses tend to be less reliable when estimated from small sample sizes [46]. Future studies with larger samples will allow factor analysis with this population. Finally, we did not evaluate the responsiveness, which is used to detect HRQOL changes while a patient’s health status changes over time and can be regarded as additional evidence of instrument validity [33]. Thus, further longitudinal studies are advised to assess responsiveness.

## Conclusions

This study is important because it is the first time the PedsQL^TM^ 3.0 Neuromuscular Module has been applied in China. Our results provide reasonable evidence that the Chinese version of the PedsQL^TM^ 3.0 Neuromuscular Module has acceptable psychometric properties. Given the potential differences between patient and proxy reports, we recommend that the pediatric patient self-report should be collected as the primary patient-reported outcome, and the parent proxy-report should be collected as a secondary patient-reported outcome. Further longitudinal studies with larger samples should be carried out to conduct factor analysis and testing responsiveness of the Chinese version scale, particularly in rural areas.

## Abbreviations

DMD: Duchenne muscular dystrophy; HRQOL: Health-related quality of life; PedsQLTM: Pediatric quality of life inventory^TM^; ICC: Intraclass correlation.

## Competing interests

The authors declare that they have no competing interests.

## Authors’ contributions

JH conceptualized and designed the study; acquired, analyzed, and interpreted the data; and drafted the manuscript. SH and LC conceptualized and designed the study, acquired data, and revised the manuscript. MK and YY conceptualized and designed the study; acquired, analyzed, and interpreted the data; and revised the manuscript. LJ conceptualized and designed the study, supervised the data analysis, and revised the manuscript. All authors read and approved the final manuscript.

## References

[B1] Emery AE Population frequencies of inherited neuromuscular diseases–a world survey Neuromuscul Disord 1991 1 1 19 29 10.1016/0960-8966(91)90039-U 1822774

[B2] Kunkel LM Hejtmancik JF Caskey CT Speer A Monaco AP Middlesworth W Colletti CA Bertelson C Müller U Bresnan M Analysis of deletions in DNA from patients with becker and duchenne muscular dystrophy Nature 1986 322 6074 73 77 10.1038/322073a0 3014348

[B3] Chakkalakal JV Thompson J Parks RJ Jasmin BJ Molecular, cellular, and pharmacological therapies for duchenne/becker muscular dystrophies FASEB J 2005 19 8 880 891 10.1096/fj.04-1956rev 15923398

[B4] Moxley RT 3rd Ashwal S Pandya S Connolly A Florence J Mathews K Baumbach L McDonald C Sussman M Wade C Practice parameter: corticosteroid treatment of duchenne dystrophy: report of the quality standards subcommittee of the American academy of neurology and the practice committee of the child neurology society Neurology 2005 64 1 13 20 10.1212/01.WNL.0000148485.00049.B7 15642897

[B5] Manzur AY Kuntzer T Pike M Swan A Glucocorticoid corticosteroids for duchenne muscular dystrophy Cochrane Database Syst Rev 2008 1 CD003725 18254031 10.1002/14651858.CD003725.pub3

[B6] Bushby K Finkel R Birnkrant DJ Case LE Clemens PR Cripe L Kaul A Kinnett K McDonald C Pandya S Diagnosis and management of duchenne muscular dystrophy, part 1: diagnosis, and pharmacological and psychosocial management Lancet Neurol 2010 9 1 77 93 10.1016/S1474-4422(09)70271-6 19945913

[B7] Bushby K Finkel R Birnkrant DJ Case LE Clemens PR Cripe L Kaul A Kinnett K McDonald C Pandya S Diagnosis and management of duchenne muscular dystrophy, part 2: implementation of multidisciplinary care Lancet Neurol 2010 9 2 177 189 10.1016/S1474-4422(09)70272-8 19945914

[B8] Moxley RT 3rd Pandya S Ciafaloni E Fox DJ Campbell K Change in natural history of duchenne muscular dystrophy with long-term corticosteroid treatment: implications for management J Child Neurol 2010 25 9 1116 1129 10.1177/0883073810371004 20581335

[B9] Young HK Lowe A Fitzgerald DA Seton C Waters KA Kenny E Hynan LS Iannaccone ST North KN Ryan MM Outcome of noninvasive ventilation in children with neuromuscular disease Neurology 2007 68 3 198 201 10.1212/01.wnl.0000251299.54608.13 17224573

[B10] Mah JK Thannhauser JE Kolski H Dewey D Parental stress and quality of life in children with neuromuscular disease Pediatr Neurol 2008 39 2 102 107 10.1016/j.pediatrneurol.2008.04.011 18639753

[B11] Iannaccone ST Hynan LS Morton A Buchanan R Limbers CA Varni JW AmSMART Group The PedsQL™ in pediatric patients with spinal muscular atrophy: feasibility, reliability, and validity of the pediatric quality of life inventory™ generic core scales and neuromuscular module Neuromuscul Disord 2009 19 12 805 812 10.1016/j.nmd.2009.09.009 19846309 PMC2796341

[B12] Davis SE Hynan LS Limbers CA Andersen CM Greene MC Varni JW Iannaccone ST The PedsQL™ in pediatric patients with duchenne muscular dystrophy: feasibility, reliability, and validity of the pediatric quality of life inventory™ neuromuscular module and generic core scales J Clin Neuromuscul Dis 2010 11 3 97 109 10.1097/CND.0b013e3181c5053b 20215981

[B13] Felder-Puig R Frey E Proksch K Varni JW Gadner H Topf R Validation of the German version of the pediatric quality of life inventory (PedsQL) in childhood cancer patients off treatment and children with epilepsy Qual Life Res 2004 13 1 223 234 15058802 10.1023/B:QURE.0000015305.44181.e3

[B14] Sprangers MA Cull A Bjordal K Groenvold M Aaronson NK The European organization for research and treatment of cancer. Approach to quality of life assessment: guidelines for developing questionnaire modules. EORTC study group on quality of life Qual Life Res 1993 2 4 287 295 10.1007/BF00434800 8220363

[B15] Patrick DL Deyo RA Generic and disease-specific measures in assessing health status and quality of life Med Care 1989 27 3 Suppl S217 S232 2646490 10.1097/00005650-198903001-00018

[B16] Varni JW Burwinkle TM Katz ER Meeske K Dickinson P The PedsQL in pediatric cancer: reliability and validity of the pediatric quality of life inventory generic core scales, multidimensional fatigue scale, and cancer module Cancer 2002 94 7 2090 2106 10.1002/cncr.10428 11932914

[B17] Varni JW Burwinkle TM Jacobs JR Gottschalk M Kaufman F Jones KL The PedsQL in type 1 and type 2 diabetes: reliability and validity of the pediatric quality of life inventory generic core scales and type 1 diabetes module Diabetes Care 2003 26 3 631 637 10.2337/diacare.26.3.631 12610013

[B18] Varni JW Burwinkle TM Rapoff MA Kamps JL Olson N The PedsQL in pediatric asthma: reliability and validity of the pediatric quality of life inventory generic core scales and asthma module J Behav Med 2004 27 3 297 318 15259457 10.1023/b:jobm.0000028500.53608.2c

[B19] Varni JW Burwinkle TM Berrin SJ Sherman SA Artavia K Malcarne VL Chambers HG The PedsQL in pediatric cerebral palsy: reliability, validity, and sensitivity of the generic core scales and cerebral palsy module Dev Med Child Neurol 2006 48 6 442 449 10.1017/S001216220600096X 16700934

[B20] Varni JW Seid M Rode CA The PedsQL: measurement model for the pediatric quality of life inventory Med Care 1999 37 2 126 139 10.1097/00005650-199902000-00003 10024117

[B21] Eiser C Morse R Quality-of-life measures in chronic diseases of childhood Health Technol Assess 2001 5 4 1 157 11262421 10.3310/hta5040

[B22] Varni JW Seid M Kurtin PS PedsQL 4.0: reliability and validity of the pediatric quality of life inventory version 4.0 generic core scales in healthy and patient populations Med Care 2001 39 8 800 812 10.1097/00005650-200108000-00006 11468499

[B23] Varni JW Limbers CA The pediatric quality of life inventory: measuring pediatric health-related quality of life from the perspective of children and their parents Pediatr Clin North Am 2009 56 4 843 863 10.1016/j.pcl.2009.05.016 19660631

[B24] Hao Y Tian Q Lu Y Chai Y Rao S Psychometric properties of the Chinese version of the pediatric quality of life inventory 4.0 Generic core scales Qual Life Res 2010 19 8 1229 1233 10.1007/s11136-010-9672-y 20473781

[B25] Florence JM Pandya S King WM Robison JD Signore LC Wentzell M Province MA Clinical trials in duchenne dystrophy. Standardization and reliability of evaluation procedures Phys Ther 1984 64 1 41 45 6361809 10.1093/ptj/64.1.41

[B26] *Scaling and scoring of the Pediatric Quality of Life Inventory^TM^ PedsQL (Updated version)* http://www.pedsql.org

[B27] PedsQL™ translation methodologyhttp://www.pedsql.org

[B28] McHorney CA Ware JE Jr Lu JF Sherbourne CD The MOS 36-item short-form health survey (SF-36): III. Tests of data quality, scaling assumptions, and reliability across diverse patient groups Med Care 1994 32 1 40 66 10.1097/00005650-199401000-00004 8277801

[B29] Cronbach LJ Coefficient alpha and the internal structure of tests Psychometrika 1951 16 3 297 334 10.1007/BF02310555

[B30] Pedhazur EJ Schmelkin LP Measurement, design, and analysis: an integrated approach 1991 Hillsdale, NJ: Erlbaum

[B31] Nunnally JC Bernstein IH Psychometric Theory 1994 3 New York: McGraw-Hill

[B32] Bartko JJ The intraclass correlation coefficient as a measure of reliability Psychol Rep 1966 19 1 3 11 10.2466/pr0.1966.19.1.3 5942109

[B33] Fayers PM Machin D Quality of life: assessment, analysis and interpretation 2000 New York: Wiley

[B34] Wilson KA Dowling AJ Abdolell M Tannock IF Perception of quality of life by patients, partners and treating physicians Qual Life Res 2000 9 9 1041 1052 10.1023/A:1016647407161 11332225

[B35] Faul F Erdfelder E Lang AG Buchner A G*Power 3: a flexible statistical power analysis program for the social, behavioral, and biomedical sciences Behav Res Methods 2007 39 2 175 191 10.3758/BF03193146 17695343

[B36] Fayers PM Hand DJ Factor analysis, causal indicators and quality of life Qual Life Res 1997 6 2 139 150 9161114 10.1023/a:1026490117121

[B37] Cohen J Statistical Power Analysis for the Behavioral Sciences 1988 2 Hillsdale, NJ: Erlbaum

[B38] McGraw KO Wong SP Forming inferences about some intraclass correlation coefficients Psychol Meth 1996 1 1 30 46

[B39] Cremeens J Eiser C Blades M Factors influencing agreement between child self-report and parent proxy-reports on the pediatric quality of life inventory 4.0 (PedsQL) generic core scales Health Qual Life Outcomes 2006 4 58 10.1186/1477-7525-4-58 16942613 PMC1564004

[B40] Varni JW Seid M Smith Knight T Burwinkle T Brown J Szer IS The PedsQL in pediatric rheumatology: reliability, validity, and responsiveness of the pediatric quality of life inventory generic core scales and rheumatology module Arthritis Rheum 2002 46 3 714 725 10.1002/art.10095 11920407

[B41] Novick M Lewis G Coefficient alpha and the reliability of composite measurements Psychometrika 1967 32 1 1 13 10.1007/BF02289400 5232569

[B42] Sprangers MA Aaronson NK The role of health care providers and significant others in evaluating the quality of life of patients with chronic disease: a review J Clin Epidemiol 1992 45 7 743 760 10.1016/0895-4356(92)90052-O 1619454

[B43] Achenbach TM McConaughy SH Howell CT Child/adolescent behavioral and emotional problems: implications of cross-informant correlations for situational specificity Psychol Bull 1987 101 2 213 232 3562706

[B44] Upton P Lawford J Eiser C Parent–child agreement across child health-related quality of life instruments: a review of the literature Qual Life Res 2008 17 6 895 913 10.1007/s11136-008-9350-5 18521721

[B45] Eiser C Morse R Can parents rate their child’s health-related quality of life? Results of a systematic review Qual Life Res 2001 10 4 347 357 10.1023/A:1012253723272 11763247

[B46] Tabachnick BG Fidell LS Using Multivariate Analysis 2001 4 Boston, MA: Allyn and Bacon

